# Use of electronic cigarettes among secondary and high school students from a socially disadvantaged rural area in Poland

**DOI:** 10.1186/s12889-016-3417-y

**Published:** 2016-08-03

**Authors:** Dorota Kaleta, Piotr Wojtysiak, Kinga Polańska

**Affiliations:** Department of Tobacco Control, Preventive Medicine Department, Medical University of Lodz, Zeligowskiego 7/9 Str., 90-752 Lodz, Poland

**Keywords:** E-cigarettes, Smoking, Tobacco, Youth, Socially disadvantaged rural areas

## Abstract

**Background:**

The use of e-cigarettes has been growing and has become a significant public health concern. Prevention of the youth access to, initiation and continuous use of e-cigarettes with special attention to vulnerable groups is a subject of a health debate. However, still little is known about characteristics of the underage e-cigarette users from different geographic and socio-economic backgrounds as well as other potential factors associated with the use of e-cigarettes, including simultaneous use with tobacco products or alcohol. The aim of the study was to investigate the prevalence and factors associated with ever and continued e-cigarette use among the secondary and high school students from a socially disadvantaged rural area in Poland.

**Methods:**

The study sample consisted of 3552 students aged 13–19 years from Piotrkowski district. The anonymous, self-administered questionnaire adapted from the Global Youth Tobacco Survey was implemented to collect relevant information. The uni- and multivariate logistic regression analyses were applied to identify factors associated with ever, current (in the previous 30 days) and continued e-cigarette use.

**Results:**

Almost 22 % of the sample reported ever e-cigarettes use and 27 % of the respondents indicated e-cigarettes use in the past month. Boys, in comparison with girls, were more likely to report current e-cigarette use (OR = 1.7; *p* < 0.001). Current e-cigarette use was strongly associated with alcohol consumption (OR = 4.3; *p* < 0.001), current (OR = 32.5; *p* < 0.001) and ever tobacco smoking (OR = 7.5; *p* < 0.001) as well as smoking parents (OR = 1.4; *p* < 0.05) and friends (OR = 4.5; *p* < 0.05). The use of e-cigarettes was also significantly associated with harm perception (*p* < 0.001). A similar pattern was observed among ever e-cigarette users. Male gender (OR = 1.4; *p* < 0.05), current tobacco smoking (OR = 3.0; *p* < 0.01) and lack of knowledge about a ban on smoking in the school (OR = 1.4; *p* < 0.05) were predictors of the continued e-cigarette use. Higher paternal education (OR = 0.5; *p* < 0.001) and perception of e-cigarettes as more harmful comparing to tobacco (OR = 0.2; *p* < 0.001) protected from the continued e-cigarette use.

**Conclusions:**

E-cigarette use is widespread in the investigated population, especially among boys, those with other risky behaviors and with smoking parents or friends. There is a need for further research and preventive policies to protect the youth from that exposure.

**Electronic supplementary material:**

The online version of this article (doi:10.1186/s12889-016-3417-y) contains supplementary material, which is available to authorized users.

## Background

Electronic cigarettes (e-cigarettes) are battery operated electronic nicotine delivery systems designed to provide nicotine in a form of an aerosol [1–3]. The wide variety of e-cigarettes – their multiple colors, shapes and flavors – is equivalent to their potential appeal for young people [4]. Additionally, even though accessibility of e-cigarettes to the youth has different legal status depending on a country, they usually fall into a grey area of jurisdiction [4–6]. As a consequence, a significant interest of the youth in e-cigarettes and an increase in their use over the recent years have been observed [4, 7–12]. For example, studies conducted in the U.S., have shown that ever and current e-cigarette use doubled among middle and high school students during 2011–2012 [8]. Similarly, a study conducted in Poland has indicated a significant increase in the current e-cigarette use among adolescents (from 5.5 % in 2010–2011 sample to 29.9 % in the 2013–2014 sample) [7, 10].

Despite a growing number of studies, still little is known about safety and health effects of e-cigarettes [5, 13]. For adolescents, the use of nicotine-containing e-liquids creates the risk of developing dependence and initiation of tobacco use. The other concern is related to the dual use (e-cigarettes and conventional cigarettes), in which individuals are exposed and addicted to higher levels of nicotine. Consequently, as they believe that by using e-cigarettes they reduce their conventional tobacco use and are getting healthier, their chances of cessation are reduced and cessation attempts delayed [14].

Prevention of the youth access to, initiation and continuous use of e-cigarettes, with special attention to vulnerable groups, is a subject of a health debate worldwide. However, still not much is known about characteristics of the underage e-cigarette users from different geographic and socio-economic backgrounds as well as other potential factors associated with the use of e-cigarettes, including simultaneous use with tobacco products or alcohol [2, 4, 7, 9, 11, 12, 14, 15]. What needs to be pointed, is the fact that majority of the existing studies in this field have focused on the samples from urban areas. At the same time, rural regions, which are more difficult to reach, have been less frequently covered.

E-cigarettes have been available on the Polish market since early 2008 [7]. According to the current regulatory framework, selling tobacco products to people under 18 is prohibited in Poland. However, e-cigarettes are not covered by legislation, and are easily accessible to the minors [6, 16]. Therefore, knowledge about risk and protective factors of e-cigarette use, especially in socially disadvantaged rural areas is of great importance so as to enable designing and implementation of appropriate preventive measures.

The aim of the study was to investigate the prevalence and factors associated with ever and current e-cigarette use, and correlates of the continued e-cigarette use among secondary and high school students (aged 13–19) from a socially disadvantaged rural area in Poland.

## Methods

### Setting of the study

During the period from November 2014 to May 2015 a cross-sectional study was conducted among secondary and high school students from Piotrkowski district, which is a socially disadvantaged rural area in central Poland (Lodzkie vivodship - administrative region of central Poland). According to the state as of 31.12.2013, there were 91,618 residents, including 45,223 men and 46,395 women, living on the premises of the district. More than 90 % of the residents of the district (82,854 individuals) were people who lived in the rural area (villages with fewer than ten thousands of residents). In 2012, in Piotrkowski district 23 % of its residents required the support of social assistance institutions due to the lack of resources to live on [17], and poverty concerned 11,867 people in 4336 families. This problem is stable and has been present for years.

Based on the data from the District Labour Office in Piotrków Trybunalski, in 2012, in total there were 4393 unemployed people registered, including 2105 people who were unemployed on a long-term basis (i.e. being without work longer than 12 months). It also needs to be pointed out that unemployment rate in the district is higher than the national unemployment rate. Simultaneously, it has to be emphasized that the rate of unemployment, in the context of the rural nature of the district, is underestimated as a social problem. It is due to the fact that in compliance with the applying regulations, the status of an unemployed cannot be granted to a person if he/she has a farm of more than 2 conversion hectares.

The analysis performed by the United Nations Development Programme (UNDP), placed Piotrkowski district at the 11th position among 30 districts of all 314 rural districts that exist in Poland, with the lowest indicators of social development. Local Human Development Index (LHDI) covering three indicators: Health Index, Education Index, Welfare Index was 25.97, including Health Index HI = 26.50, whereas the discussed indicators for Lodzkie voivodeship were, respectively: 39.28 and 31.48 [18]. In the context of the wealth of districts of Lodzkie voivodeship, Piotrkowski district has very poor situation and takes the low (20^th^) position among 24 districts, and in the national classification it is 360th among 380 units in the local government. The discussed indicators got worse in 2010 compared to year 2007 and the position of the district was by 5 positions lower. The above data indicate a low level of socio-economic development of Piotrkowski district.

### Study design and population

This cross-sectional study was conducted based on the questionnaire adapted from the Global Youth Tobacco Survey (GYTS) recommended by the World Health Organization (WHO) and the Center for Disease Control and Prevention (CDC) for systematical monitoring of the youth tobacco use. Data collection was supervised and coordinated by the principal investigator and 10 field supervisors.

Before the study commenced, approvals of the Education Management Centre in Piotrkowski district and of directors of educational institutions where the research was to be carried out were obtained. All of the 16 secondary and 5 high schools in Piotrkowski district invited to participate in the project agreed to take part in the study.

In total, 2997 students attending secondary schools and 1053 students attending high schools in Piotrkowski district were invited to participate in the study. In the group of the respondents attending secondary schools, the filled in questionnaires were returned by 2645 (88.3 %) teenagers, whereas among those from high schools they were filled in and returned by 907 (86.1 %) individuals.

### Questionnaire and study measures

An anonymous, self-administrated questionnaire, filled in by the students during regular classes, consisted of 84 questions, including core questions from original GYTS questionnaire, and additional items concerning the nature and legislation applicable in Poland. The questionnaire was divided into the following sections: 1) prevalence of cigarette use (including tobacco and e-cigarettes), 2) environmental tobacco smoke (ETS) exposure, 3) knowledge and attitudes towards cigarette use and existing legislation in this field, 4) willingness and attempts to quit smoking, 5) education in the field of tobacco use and tobacco control interventions obtained during regular classes, 6) accessibility of tobacco products, 7) pro- and anti-tobacco advertising, and 8) demographic and social data.

The use of e-cigarettes was assessed via the question: “Have you ever (even once) tried e-cigarettes (electronic cigarettes)?” Those who responded “no” to this question were considered as never e-cigarette users. Ever e-cigarette users were defined as those who have tried e-cigarettes but did not use them within the month preceding the study. Those who have used e-cigarettes at least once during the past 30 days were classified as current e-cigarette users. Similar procedures were used to categorize traditional cigarette users. In terms of alcohol consumption, the adolescents were divided into three categories: non-drinkers, moderate and binge drinkers (with the two last categories combined in the logistic regression analysis). Question on parents’ smoking was asked separately and the respondents were categorized as children of neither smokers vs. children of one or both parents being smokers. Smoking friends were divided into non, some, most of or all.

Socio-demographic variables covered: gender, age, school grade (secondary or high school), parental education (high: over 12 years of education, medium: 9–12 years of education, low: 9 years or less), and money for a student’s own expenses available per month (≤100 PLN, >100 PLN).

In order to assess knowledge and awareness of e-cigarettes as well as the attitude towards their safety several questions were used. Harm perception item was adopted from Smith et al. [19]. Adolescents were asked: “Compared with regular cigarettes how harmful do you think e-cigarettes are”? with the possible answers: just as harmful, less harmful or more harmful. The youth’s perception of traditional cigarettes as harmful to health was also assessed. Finally, the respondents were asked if their school had any rules regarding e-cigarette use and about a tobacco smoking ban in their school and in the school area.

### Statistical analysis

The data were entered into the Excel data analysis software on a daily basis by field investigators and then submitted to a supervisor. Once the data collection process had been completed, 5 % of the records were randomly checked to confirm that they were clearly recorded, complete and consistent across responses. Data set is provided in (Additional file 1). The univariate and multivariate logistic regression analyses with the results being presented as odds ratios (OR) with 95 % confidence intervals (95 % CI) were applied to the study correlates of ever and current e-cigarette use among the youth. First, we ran the univariate logistic regression analyses with ever and current e-cigarette use as dependent variables and demographic, risky behaviors (tobacco smoking and alcohol) and factors describing knowledge of e-cigarettes as independent variables. Next, we performed the multiple logistic regression analyses that included all variables significantly associated with e-cigarette use in any of the univariate models (*p* ≤ 0.05).

Analogous analysis was conducted to explore factors associated with the continued e-cigarette use. For this purpose, the never e-cigarette users were excluded and the predictive factors were tested to determine what may influence e-cigarette use on an on-going basis (the current users *n* = 975 vs. the ever users *n* = 770).

The STATISTICA Windows XP version 10.0 program was used to carry out the statistical analyses.

## Results

### Characteristics of the study population

Information on the sample demographics, risky behaviors (tobacco smoking and alcohol consumption), knowledge and awareness of e-cigarettes as well as the attitude towards their safety are presented in Table 1. Boys represented 56 % of our study sample. Most of the respondents (75 %) were attending secondary schools. Three-quarters of the youth indicated alcohol consumption and 30 % of them current tobacco smoking (similar percentage of the students were ever smokers). About 39 % of the youth thought that e-cigarettes were just as harmful as traditional cigarettes, and half of them considered e-cigarettes as less harmful. Almost 60 % of the study participants indicated that school did not have any rules restricting e-cigarette use.

**Table 1:** Prevalence of e-cigarette use by socio-demographic characteristics in the adolescents from socially disadvantaged rural area in Poland

Characteristic	Never e-cigarette users *n* = 1807 (50.9 %)		Ever e-cigarette users *n* = 770 (21.7 %)		Current e-cigarette users *n* = 975 (27.4 %)	
	n (%)	95 % CI	n (%)	95 % CI	n (%)	95 % CI
Gender						
Male	922 (46.0)	43.8–48.2	437 (21.8)	20.0–23.6	645 (32.2)	30.2–34.3
Female	885 (57.2)	54.7–59.7	333 (21.5)	19.5–23.6	330 (21.3)	19.3–23.3
Age (years)						
13	493 (61.9)	59.8–64.0	135 (16.9)	14.3–19.5	169 (21.2)	18.4–24.0
14	485 (52.4)	49.2–55.6	204 (22.1)	19.4–24.8	236 (25.5)	22.7–28.3
15	443 (48.0)	44.8–51.2	198 (21.4)	18.7–24.0	282 (30.6)	27.6–33.6
16	93 (37.5)	31.5–43.5	62 (25.0)	19.6–30.4	93 (37.5)	31.5–43.5
17	97 (42.7)	36.3–49.1	59 (26.0)	20.3–31.7	71 (31.3)	25.3–37.3
18	79 (45.4)	38.0–52.8	42 (24.1)	17.7–30.5	53 (30.5)	23.7–37.3
19	117 (45.4)	39.3–51.5	70 (27.1)	21.7–32.5	71 (27.5)	22.1–33.0
School grade						
Secondary	1421 (53.7)	51.8–55.6	537 (20.3)	18.8–21.8	687 (26.0)	24.3–27.7
High	386 (42.6)	39.4–45.8	233 (25.7)	22.9–28.5	288 (31.7)	28.7–34.7
Father education						
Low	1007 (46.1)	44.0–48.2	466 (21.4)	19.7–23.1	710 (32.5)	30.5–34.5
Medium	442 (56.2)	52.7–59.7	178 (22.6)	19.7–25.5	166 (21.1)	18.2–24.0
High	358 (61.4)	57.5–65.4	126 (21.6)	18.3–24.9	99 (17.0)	13.9–20.0
Mother education						
Low	773 (44.4)	42.1–46.7	416 (23.9)	21.9–25.9	552 (31.7)	29.5–33.9
Medium	452 (45.5)	42.4–48.6	221 (22.2)	19.6–24.8	321 (32.3)	29.4–35.2
High	582 (71.2)	68.1–74.3	133 (16.3)	13.8–18.8	102 (12.5)	10.2–14.8
Money available per month						
≤ 100 PLN	1056 (44.9)	42.9–46.9	527 (22.4)	20.7–24.1	769 (32.7)	30.8–34.6
> 100 PLN	751 (62.6)	59.9–65.3	243 (20.2)	18.0–22.6	206 (17.2)	15.1–19.3
Alcohol use						
Non-drinker	696 (78.8)	76.1–81.5	78 (8.8)	6.9–10.7	109 (12.3)	10.1–14.5
Moderate	554 (89.9)	87.5–92.3	30 (4.9)	3.2–6.6	32 (5.2)	3.4–7.0
Binge	557 (27.1)	25.2–29.0	662 (32.2)	30.2–24.2	834 (40.6)	38.5–42.7
Tobacco cigarette smoking status of the adolescents						
Never tobacco smoker	1197 (84.0)	82.1–85.9	143 (10.0)	8.4–11.6	85 (6.0)	4.8–7.2
Ever tobacco smoker	436 (40.2)	37.4–43.2	358 (33.1)	30.3–35.9	289 (26.7)	24.1–29.3
Current tobacco smoker	174 (16.7)	14.4–19.0	269 (25.8)	23.1–28.5	601 (57.6)	54.6–60.6
Parental smoking						
None	1083 (58.6)	56.4–60.9	349 (18.9)	17.1–20.7	415 (22.5)	20.6–24.4
One or both parents	724 (42.5)	40.1–44.8	421 (24.7)	22.6–26.7	560 (32.8)	30.6–35.0
Friends smoking						
None	496 (74.0)	70.7–77.3	84 (12.5)	10.0–15.0	90 (13.4)	10.8–16.0
Some	1029 (58.3)	56.0–60.6	385 (21.8)	19.9–23.7	350 (19.8)	17.9–21.7
Most or all	282 (25.2)	22.7–27.7	301 (26.9)	24.4–29.4	535 (47.9)	45.0–50.7
Perception that tobacco smoking is harmful to health						
No	126 (32.0)	27.4–36.6	96 (24.4)	20.2–28.6	172 (43.6)	38.8–48.6
Yes	1681 (53.2)	51.5–54.9	674 (21.3)	19.9–22.7	803 (25.4)	23.9–26.9
E-cigarette harm perception						
As harmful	722 (52.1)	49.5–54.7	260 (18.7)	16.7–20.9	405 (29.2)	26.8–31.6
Less harmful	964 (51.4)	49.1–53.7	384 (20.5)	18.7–22.3	527 (28.1)	26.1–30.1
More harmful	121 (41.7)	36.0–47.4	126 (43.5)	37.8–49.1	43 (14.8)	13.9–22.9
School has the rules restricting e-cigarettes use						
Yes	790 (54.9)	52.3–57.5	310 (21.5)	19.4–23.6	340 (23.6)	21.4–25.8
No	1017 (48.2)	46.1–50.3	460 (21.8)	20.0–23.6	635 (30.1)	28.1–32.1
Smoking ban in a school building and in the school area						
Yes in all places	1346 (54.0)	52.0–56.0	541 (21.7)	20.1–23.3	607 (24.3)	22.6–26.0
Yes in a school building but not in the school area	111 (45.9)	39.6–52.2	59 (24.4)	19.0–29.8	72 (29.8)	24.0–35.6
No	350 (42.9)	39.5–46.3	170 (20.8)	18.0–23.6	296 (36.3)	33.0–39.6

### Prevalence of e-cigarette use

Almost 22 % of the sample reported ever e-cigarette use, and 27 % of the respondents indicated e-cigarette use in the past month (Table 1). Ever e-cigarette use was equally declared by the boys and girls (22 %), whereas current e-cigarette use was declared more frequently by the male (32 %) than female gender (21 %). Higher percentages of the ever and the current e-cigarette users were observed among students from the high schools (26 and 32 % respectively) than from the secondary schools (20 and 26 % respectively). Those whose parents had the highest educational level indicated current e-cigarette use less frequently than those whose parents were in the lowest educational level category (for mothers 13 % vs. 32 %; for fathers 17 % vs. 33). A similar percentage was observed for those who had more money available for their own expenses comparing to the group with smaller amount of money (17 % vs. 33 %). Those patterns were not observed among ever e-cigarette users. Among the students who indicated no alcohol consumption there were fewer ever and current e-cigarette users compared to the group indicating alcohol consumption. Among the never tobacco users, 6 % declared current and 10 % ever e-cigarette smoking. Dual use (current tobacco smoking and e-cigarette use) was indicated by 58 % of the respondents. In the group of the youth whose parents and friends smoked tobacco, e-cigarettes were indicated more frequently than in the group with non-smoking relatives. When asked how harmful e-cigarettes were compared to regular cigarettes among those who indicated no differences in harmful effects between the two kinds of products, about 19 % were ever-cigarette users and 29 % used e-cigarettes currently. In the group of teenagers who thought that e-cigarettes were more harmful than traditional cigarettes 44 % were ever and 15 % current e-cigarette users. Those who indicated that their school had no rules regarding e-cigarette, used e-cigarettes more frequently than the group indicating existence of such regulations in their school. This pattern was not observed in the group of ever e-cigarette users.

### Factors associated with ever and current e-cigarette use

Table 2 displays results of the logistic regression analyses examining factors associated with ever and current e-cigarette use. Based on the univariate analysis, the following predictors of the current e-cigarette use were identified: male gender (*p* < 0.001), older age (*p* < 0.001), alcohol consumption (*p* < 0.001), ever and current tobacco smoking (*p* < 0.001), parental and friends smoking status (*p* < 0.001), and opinion indicating no restrictions regarding e-cigarettes and tobacco cigarettes use in the school (*p* < 0.001). Higher parental education (*p* < 0.001), more money available for own expenses (*p* < 0.001) and perception of e-cigarettes as more harmful compared to traditional cigarettes (*p* = 0.01) were protective when considering current e-cigarette use. A similar pattern was observed among the ever e-cigarette users.

**Table 2:** Correlates of ever and current e-cigarette use in the adolescents from socially disadvantaged rural area in Poland

	Ever e-cigarette users		Current e-cigarette users	
Characteristic	Unadjusted OR (95 % CI)	Adjusted OR (95 % CI)	Unadjusted OR (95 % CI)	Adjusted OR (95 % CI)
Gender				
Male	1.3 (1.1–1.5)**	1.3 (1.0–1.6)**	1.9 (1.6–2.2)*	1.7 (1.4–2.2)*
Female	1.0 (Ref.)	1.0 (Ref.)	1.0 (Ref.)	1.0 (Ref.)
Age (years)	1.1 (1.1–1.2)*	0.9 (0.8–1.0)	1.1 (1.1–1.2)*	0.9 (0.8–1.0)
School grade				
Secondary	1.0 (Ref.)	1.0 (Ref.)	1.0 (Ref.)	1.0 (Ref.)
High	1.6 (1.3–1.9)*	1.1 (0.7–1.7)	1.5 (1.3–1.8) *	1.0 (0.6–1.7)
Father education				
Low	1.0 (Ref.)	1.0 (Ref.)	1.0 (Ref.)	1.0 (Ref.)
Medium	0.9 (0.7–1.1)	1.5 (1.1–2.0)**	0.5 (0.4–0.7)*	0.9 (0.6–1.2)
High	0.8 (0.6–0.9)**	1.1 (0.8–1.6)	0.4 (0.3–0.5)*	0.6 (0.4–0.9)**
Mother education				
Low	1.0 (Ref.)	1.0 Ref.	1.0 (Ref.)	1.0 (Ref.)
Medium	0.9 (0.7–1.1)	0.8 (0.6–1.0)	1.0 (0.8–1.2)	0.9 (0.7–1.2)
High	0.4 (0.3–0.5)*	0.5 (0.4–0.7)*	0.3 (0.2–0.3)*	0.5 (0.3–0.7)*
Money available per month				
≤ 100 PLN	1.0 (Ref.)	1.0 (Ref.)	1.0 (Ref.)	1.0 (Ref.)
> 100 PLN	0.7 (0.5–0.8)*	1.0 (0.8–1.3)	0.4 (0.3–0.5)*	0.8 (0.6–1.1)
Alcohol use				
Non-drinker (a)	1.0 (Ref.)	1.0 (Ref.)	1.0 (Ref.)	1.00 Ref.
Moderate & Binge	5.6 (4.3–7.2)*	5.3 (4.0–7.1)*	5.0 (4.0–6.2)*	4.3 (3.2–5.7)*
Tobacco cigarette smoking status of the adolescents				
Never tobacco smoker	1.0 (Ref.)	1.0 (Ref.)	1.0 (Ref.)	1.0 (Ref.)
Ever tobacco smoker	6.9 (5.5–8.6)*	6.7 (5.2–8.7)*	9.3 (7.2–12.2)*	7.5 (5.5–10.1)*
Current tobacco smoker	12.9 (10.0–16.8)*	9.8 (7.2–13.3)*	48.6 (36.8–64.3)*	32.5 (23.2–45.5)*
Parental smoking				
None	1.0 (Ref.)	1.0 (Ref.)	1.0 (Ref.)	1.0 (Ref.)
One or both parents	1.8 (1.5–2.1)*	1.4 (1.1–1.7)**	2.0 (1.7–2.4)*	1.4 (1.1–1.8)**
Friends smoking				
None	1.0 (Ref.)	1.0 (Ref.)	1.0 (Ref.)	1.0 (Ref.)
Some	2.2 (1.7–2.9)*	1.4 (1.0–1.9)**	1.9 (1.5–2.4)*	1.5 (1.1–2.2)**
Most or all	6.3 (4.8–8.4)*	2.3 (1.7–3.3)*	10.5 (8.0–13.7)*	4.5 (3.1–6.5)**
Perception that tobacco smoking is harmful to health				
No	1.9 (1.4–2.0)*	1.9 (1.3–2.7)*	2.9 (2.2–3.7)*	3.2 (2.2–4.7)*
Yes	1.0 (Ref.)	1.0 (Ref.)	1.0 (Ref.)	1.0 (Ref.)
E-cigarette harm perception				
As harmful	1.0 (Ref.)	1.0 (Ref.)	1.0 (Ref.)	1.0 (Ref.)
Less harmful	1.1 (0.9–1.3)	1.8 (1.4–2.2)*	1.0 (0.8–1.1)	2.1 (1.7–2.7)*
More harmful	2.9 (2.2–3.9)**	2.7 (1.9–3.8)*	0.6 (0.4–0.9)**	0.3 (0.2–0.6)*
School has the rules restricting e-cigarettes use				
Yes	1.0 (Ref.)		1.0 (Ref.)	1.0 (Ref.)
No	1.2 (1.0–1.4)		1.5 (1.2–1.7)*	0.9 (0.7–1.2)
Smoking ban in a school building and in the school area				
Yes in all places	1.0 (Ref.)		1.0 (Ref.)	1.0 (Ref.)
Yes in a school building but not in the school area	1.3 (1.0–1.8)		1.4 (1.1–2.0)**	0.7 (0.5–1.2)
No	1.2 (1.0–1.5)		1.9 (1.6–2.3)*	1.0 (0.8–1.4)

* ≤ 0.001

** < 0.05

The multivariate analyses indicated that males were more likely to report current and ever e-cigarette use than females (OR = 1.7 and OR = 1.3; *p* < 0.05 respectively) (Table 2). E-cigarette use was strongly associated with alcohol consumption (OR = 5.3 among the ever e-cigarette and OR = 4.3 among the current e-cigarette users; *p* < 0.001), tobacco smoking including both current (OR = 9.8 among the ever e-cigarette and OR = 32.5 among the current e-cigarette users; *p* < 0.001) and ever tobacco smoking (OR = 6.7 among the ever e-cigarette and OR = 7.5 among the current e-cigarette users; *p* < 0.001) as well as parental (OR = 1.4; *p* < 0.05 for both the ever and current e-cigarette users) and friends smoking (OR = 2.3 among the ever e-cigarette and OR = 4.5 among the current e-cigarette users; *p* < 0.05).

The use of e-cigarettes was also significantly associated with harm perception (*p* < 0.05). Those who thought that e-cigarettes were less harmful than traditional cigarettes had a higher risk of ever and current e-cigarette use (OR = 1.8; and OR = 2.1; *p* < 0.001 respectively). In the group of the youth who indicated that e-cigarettes were more harmful when compared to tobacco cigarettes, the risk of being an ever e-cigarette user was higher (OR = 2.7; *p* < 0.001) and current smoking lower (OR = 0.3; *p* < 0.001) compared to those who indicated a similar harmful effect of the two discussed products. Finally, a higher maternal educational level was protective when considering ever and current e-cigarette use (OR = 0.5; *p* < 0.001). Knowledge about school regulations regarding e-cigarette and tobacco cigarette use in the school did not have a significant impact on e-cigarette pattern.

### Factors associated with continued e-cigarette use

Of the included demographic variables, risky behaviors and knowledge, and awareness of e-cigarettes, male gender (OR = 1.4; *p* < 0.05), current tobacco smoking (OR = 3.0; *p* < 0.01), and lack of knowledge about a ban on smoking in the school (OR = 1.4; *p* < 0.05) were predictors of the continued e-cigarette use (Table 3). Higher parental education (OR = 0.5; *p* < 0.001) and perception of e-cigarettes as more harmful compared to tobacco cigarettes (OR = 0.2; *p* < 0.001) protected from the current e-cigarette use.

**Table 3:** Correlates of the continued e-cigarette use in the adolescents from socially disadvantaged rural area in Poland

Characteristic	Unadjusted OR (95 % CI)	Adjusted OR (95 % CI)
Gender		
Male	1.5 (1.2–1.8)*	1.4 (1.1–1.8)**
Female	1.0 (Ref.)	1.0 (Ref.)
Age (years)	1.0 (0.9–1.0)	
School grade		
Secondary	1.0 (Ref.)	
High	1.0 (0.8–1.2)	
Father education		
Low	1.0 (Ref.)	1.0 (Ref.)
Medium	0.6 (0.5–0.8)*	0.7 (0.5–0.9)**
High	0.5 (0.4–0.7)*	0.5 (0.4–0.7)*
Mother education		
Low	1.0 (Ref.)	1.0 Ref.
Medium	1.1 (0.9–1.4)	1.0 (0.7–1.3)
High	0.6 (0.4–0.8)*	0.8 (0.6–1.1)
Money available per month		
≤ 100 PLN	1.0 (Ref.)	1.0 (Ref.)
> 100 PLN	0.6 (0.5–0.7)*	0.8 (0.6–1.0)
Alcohol use		
Non-drinker (a)	1.0 (Ref.)	
Moderate & Binge	0.9 (0.7–1.2)	
Tobacco cigarette smoking status of the adolescents		
Never tobacco smoker	1.0 (Ref.)	1.0 (Ref.)
Ever tobacco smoker	1.4 (1.0–1.9)	1.0 (0.7–1.4)
Current tobacco smoker	3.8 (2.8–5.1)*	3.0 (2.1–4.2)*
Parental smoking		
None	1.0 (Ref.)	
One or both parents	1.1 (0.9–1.4)	
Friends smoking		
None	1.0 (Ref.)	1.0 (Ref.)
Some	0.9 (0.6–1.2)	0.9 (0.6–1.3)
Most or all	1.7 (1.2–2.3)**	1.4 (0.9–2.0)
Perception that tobacco smoking is harmful to health		
No	1.5 (1.2–2.0)**	1.2 (0.8–1.6)
Yes	1.0 (Ref.)	1.0 (Ref.)
E-cigarette harm perception		
As harmful	1.0 (Ref.)	1.0 (Ref.)
Less harmful	0.9 (0.7–1.1)	0.9 (0.7–1.2)
More harmful	0.2 (0.1–0.3)*	0.2 (0.1–0.3)*
School has the rules restricting e-cigarettes use		
Yes	1.0 (Ref.)	1.0 (Ref.)
No	1.3 (1.0–1.5)**	1.2 (1.0–1.5)
Smoking ban in a school building and in the school area		
Yes in all places	1.0 (Ref.)	1.0 (Ref.)
Yes in a school building but not in the school area	1.1 (0.8–1.6)	0.9 (0.6–1.3)
No	1.6 (1.2–1.9)*	1.4 (1.0–1.8)**

* ≤ 0.001

** < 0.05

## Discussion

The study indicates that a high proportion of the youth from a socially disadvantaged rural area of Poland uses e-cigarettes; with male gender, alcohol consumption and tobacco smoking being predictors of the use of these products. The use of e-cigarettes was also significantly associated with harm perception and parental as well as friends smoking status. In addition, male gender, current tobacco smoking and lack of knowledge about a ban on smoking in the school were predictors of the continued e-cigarette use, whereas, higher parental education as well as perception of e-cigarettes as more harmful than tobacco cigarettes were protective factors of the continued e-cigarette use.

We found that almost 22 % of the adolescents from a socially disadvantaged rural area reported ever e-cigarette use and 27 % of them indicated e-cigarette use in the month preceding the study. These results are in line with the recent study conducted in Poland by Goniewicz et al. [10], where current e-cigarette use was declared by about 30 % of the youth population. Taking into consideration accessibility of tobacco products (including access via Internet) and exposure to e-cigarette advertisements, it can be suspected that in urban areas e-cigarette use may be even higher [20]. When comparing the prevalence of e-cigarette use between the countries, it is crucial to be aware of the year of investigation as the popularity of those products, especially among young people, has increased rapidly. It has been proven by existing research in this field. As an example, based on the data from the survey mentioned above, current e-cigarette use in the 2010–2011 sample was 5.5 %, whereas in the 2013–2014 sample it was almost six times higher [7, 10]. Other studies have also shown that ever and current e-cigarette use among students has increased significantly in the recent years [1, 8, 9, 21, 22].

It should be underlined that cigarette smoking as well as e-cigarette use is a learned behavior, which passes through various stages, namely: preparation, initiation, experimentation, regular and long-term use and addition [23]. Low prices of e-cigarettes, easy access to them as well as marketing activities and the fashion, all constitute factors that significantly contribute to e-cigarette initiation and continuous use [2]. It is also worth to mention that e-cigarette industry has a significant online presence, through which e-cigarettes have been promoted as both a safer alternative to cigarette smoking and a dual use product in place where smoking is not allowed [2]. The meaning of e-cigarettes in adolescents’ everyday life may be similar to that of conventional cigarette use since they share similar features [4]. In addition e-cigarettes may appeal to adolescents with novelty-seeking or sensation-seeking characteristics [4]. Another interpretation could be that e-cigarettes appeal to young people in the process of forming a smoker identity – a known risk factor for smoking escalation [4].

In line with the studies conducted in the U.S., Canada and Europe, gender was found to be a predictor of e-cigarette use with males being more likely to have tried this product [2, 4, 7, 14, 15, 24–29]. This may be due to socio-cultural determinants and it can also indicate to what extent young people are influenced and susceptible to marketing activities and current trends. In addition, boys may be “early adopters” of technology, and perhaps, they can get attracted to e-cigarettes earlier due to their novelty [4].

While the use of e-cigarettes may be driven by the desire to quit smoking in the population of older, more established smokers, the findings suggest that intention to quit does not play a crucial role in the e-cigarette use among the youth [30]. In our study, among the never tobacco users 6 % declared current and 10 % ever e-cigarette use. Adolescence is the time when individuals often experiment with and initiate the use of some substances [31]. This can create a risk of developing dependence and initiation of tobacco use. In addition, dual use (current tobacco smoking and e-cigarette use) was indicated by 58 % of our study respondents. Similarly, in other studies the higher risk of e-cigarette use was observed among daily tobacco smokers [2, 4, 7, 15, 24, 30]. This can result in a situation when the individuals are exposed and addicted to higher levels of nicotine and thus, it reduces their chances of cessation.

We also identified alcohol consumption as a risk factor for e-cigarette use, which strengthens the concept formulated as a result of the studies, conducted both in the adults and the youth, that e-cigarettes may be an element of multiple product use – users of other products, such as tobacco, alcohol, have greater odds of e-cigarette use [9, 15, 30].

Problem behavior theory suggests that risky behaviors cluster because they serve the same purpose socially, developmentally and psychologically or are a manifestation of similar underlying factors [12, 32, 33]. Alternatively, the gateway and reverse gateway theories suggest that adolescent use of one substance increases the likelihood of using other substances [34].

Additionally, sensation seeking, or the need for new, different, or complex sensations and experiences, and the willingness to take risks to achieve them, associate with adolescent substance use and may increase e-cigarette experimentation [35].

Based on our assessment, similarly to the study by Goniewicz and Zielinska-Danch [7], the adolescents who had parents and friends who smoked were more likely to use e-cigarettes than those with no smoking persons in their family and surrounding. It is proven that people tend to choose their friends based on shared characteristics, including tobacco smoking or e-cigarette use [36]. However, having close friends who use these products does not need to mean that they caused the person to do so. On the other hand, strong commitment not to smoke or use an e-cigarette if offered is crucial as a protective factor for not taking up such a behavior [37, 38].

In our analysis those who perceived e-cigarettes as less harmful than tobacco cigarettes were more likely to use e-cigarettes. This indicates that a favorable perception in terms of harm, benefits of use and general appeal (i.e. flavors and colors) of novel products are related to their attractiveness, especially among the youth [15, 39, 40]. In addition, harm perception can express familiarity with the product in such a way that individuals who have not tried e-cigarettes might not have a full understanding of the risk and benefits associated with their use [30]. In this context, more research evaluating health effects of e-cigarettes and their impact on young children motivation to start using traditional cigarettes is needed. Educational activities in school curriculum increasing young people’s awareness in this area also need to be pointed.

We also evaluated correlates of the continued e-cigarette use. Among a variety of factors, male gender, current tobacco smoking and the lack of knowledge about a ban on smoking in the school predispose the youth to the continued e-cigarette use. Similar results, although conducted on a smaller sample size (which can limit the level of significance), have been observed by Babineau et al. [24]. This population should be the target group for public health action.

The current analysis has several strengths. The study has been conducted in a socially disadvantaged rural district of Poland which, as located in a remote and difficult to reach area, is usually less frequently covered by national representative surveys and preventive actions including tobacco control measures. The fact that all the secondary and high schools in Piotrkowski district were covered by the study, and a high response rate (above 85 %) constitute other significant advantages of the current assessment.

Limitations of the study also need to be pointed. Firstly, all estimates in our assessment were based on self-reports, which might be affected by reporting bias. However, the study results are similar to the previous data for the youth population in Poland, which proved reliability of the obtained results [10]. Secondly, as the study was developed to investigate different forms of tobacco or nicotine products and it was not specifically dedicated to e-cigarettes, we did not assess if the youth used e-cigarettes continuously or occasionally, or only experimented with them within the period covered by the study. In addition, there are no data about e-cigarette smoking by friends and family members of the respondents. Our assessments indicated that parents and friends smoking traditional cigarettes are significant predictors of e-cigarette use. This can be also the case when considering e-cigarette use as social influence is a very strong driver of the initiation and habituation of health behaviors. Finally, we do not have the detailed data allowing to assess their willingness to quit tobacco smoking and more psychologically oriented motivation for using e-cigarettes.

Despite the limitations, the current study provides a valuable insight into the prevalence, patterns and correlates of e-cigarette use in the adolescents from a socially disadvantaged rural area of Poland.

## Conclusions

The study indicates the widespread use of e-cigarettes among the youth from a socially disadvantaged rural district in Poland. The study findings underscore the need for further research assessing whether e-cigarettes may serve as a starter product for nonsmokers, or as the alternative or cessation measure for tobacco. Moreover, motives and risk factors for dual use of e-cigarettes and tobacco cigarettes still remain not resolved. The impact of e-cigarettes promotion and advertising, on one hand, and of educational campaigns, on the other, on the use of such products among the youth needs to be evaluated. Finally, more research is needed to fully understand safety and health effects of e-cigarette use.

## Abbreviations

95 % CI, 95 % confidence interval; CDC, Center for Disease Control and Prevention; E-cigarettes, electronic cigarettes; ETS, environmental tobacco smoke; GYTS, Global Youth Tobacco Survey; OR, odds ratio; WHO, World Health Organization.

## Additional file

Additional file 1: Data set GYTS. (CSV 792 kb)
